# Supplementary material for: Impact of improved stove intervention on infant acute respiratory infections: Results from a randomized trial in Northwest Ethiopia
Source: PLoS One. 2026 Jan 6;21(1):e0340588. doi: 10.1371/journal.pone.0340588 (PMC12774370; doi:10.1371/journal.pone.0340588)
Supplement: Attachment — Submitted filename: Response to Reviewers.docx [file pone.0340588.s002.docx]

**Title**: Impact of Improved Stoves on Infant Acute Respiratory Infections: A Randomized Controlled Trial in Northwest Ethiopia

**A point-by-point response to reviewers’ questions and suggestions**

We appreciate the reviewers’ time in making detailed constructive comments and suggestions which have greatly helped to strengthen this manuscript. Below is our point-by-point response to each comment. We hope the revised manuscript captures better the study’s rigor, improves the quality of the writing and content, and appeals to a broader audience.

**Reviewer #1**

**Reviewer point # 1**: Given that the sample size is fixed, please present what detectable difference was possible for given alpha and power. Please also account for clustering in this calculation.

**Authors’ response # 1:** Thank you for raising this important point. We agree that the **minimum detectable** difference, is the appropriate statistical measure for assessing the precision of a completed study with a fixed sample size. Since the sample size for this follow-up was fixed based on the live births from the parent trial, we employed a minimum detectable effect size calculation. This approach reverses the standard power analysis question of 'how many participants are required to detect a difference?' to answer the question **'given our final sample size (**N=343 **live births, with** n=176 **in the intervention group and** n=167 **in the control group), what is the smallest true difference between the two groups that the study was adequately powered to detect?'**

Observed prevalence in control arm was 13% and the sample size for intervention group was 176 and for that of control group was 167. With the assumption of 5% two-sided Significance level and 80% statistical power, the study will detect an absolute difference of at least 10.2 percentage points in the proportion of infants experiencing at least one ARI episode over the 6-month period. But, for the intervention to be statistically significant at the p < 0.05 level with 80% power, the prevalence in the intervention group would need to be 13.0% - 10.2% = **2.8%** or lower. The observed difference was 13.0% - 10.7% = **2.3** percentage points. Since the observed difference (2.3%) is significantly smaller than the minimum detectable difference (10.2%), the study was statistically underpowered to detect the effect that was seen (see page 4, lines 113-117).

**Reviewer point # 2:** Please describe the details of randomization

**Authors response # 2:** Thank you for your comment. We have accepted it, and corrections have been made accordingly (see pages 5, lines 133-142). The randomization procedure is also clearly presented in the CONSORT (Consolidated Standards of Reporting Trials) flow diagram shown in Figure 2.

**Reviewer point # 3**: When did the neonatal deaths occur? These should not be excluded. Was the cause of death respiratory infection?

**Authors’ response # 3:** Thank you for your comment. All neonatal deaths occurred prior to the first scheduled study visit. Since these infants were deceased upon initial contact, they could not be included in the primary analysis (intervention exposure or follow-up data). The total number of deaths was very small (n=3; 1 control, 2 intervention) and their exclusion, occurring before data collection and analysis, does not impact the internal validity or conclusions drawn from the remaining data. The cause of death was not acute respiratory infection but was attributed to birth asphyxia and sepsis.

**Reviewer point # 4:** What correlation structure was used for GEE? Were robust standard errors used? Please report these details in the statistical analysis section. Generally more details are needed. Please write out the model completely for me - including what the outcome looks like.

**Authors’ response # 4:** We appreciate your request for additional detail regarding the Generalized Estimating Equations (GEE) approach. We agree that these specifics are essential for reproducibility and transparency, and we have now fully elaborated these details in the statistical analysis section of the manuscript. The GEE model was fitted using an Exchangeable Working Correlation Structure. This structure was chosen because our data involves repeated measurements of the outcome taken at fixed, scheduled intervals (visits) across all participants. The exchangeable structure assumes that the correlation between any two measurements taken on the same participant is constant, regardless of the time interval between them. While we acknowledge that an ARI structure might also be plausible for time-based data, the Exchangeable structure offered a more parsimonious fit and did not significantly alter the inference compared to more complex structures.

Regarding the use of robust standard errors, robust (empirical) standard errors were used in the GEE model. The robust standard errors were employed to account for any potential misspecification of the working correlation structure. These standard errors provide consistent estimates of the variance even if the specified exchangeable correlation structure is incorrect, thereby ensuring the validity of our *p*-values and confidence intervals.

The GEE model was used to analyze the effect of the intervention on the repeatedly measured outcome (the incidence of acute respiratory infection) over the course of the follow-up period.

**The full GEE model used is specified as follows**:

g (E[Yij/ Xij)= β0+β1*intervention +β2* visit j +β3 * (intervention I * visit j) +Xij

Where

- i indexes the participant (i=1,…., 343)
- j indexes the study visit (time point) where measurement was taken (j=1,…, total visit which was 3

The outcome variable, Yij, represents the **binary status of acute respiratory infection** for participant i at visit j. Yij = 1 if the participant was diagnosed with an acute respiratory infection at visit j. Yij = 0 otherwise. Therefore, the **distribution** specified for the GEE model was **binomial.** Given the binomial distribution, the Link Function used was the logit function.

βo is the intercept (log-odds of the outcome in the control group at the baseline visit). The intervention is binary indicator for the treatment arm (1 for intervention group and 0 for control group). The coefficient β1 represents the difference in the log-odds of the outcome between the intervention and control groups at baseline (j=1). Whereas Visit j is a categorical or continuous variable representing the time point/visit number. The coefficient β2 represents the change in log-odds of the outcome per unit increase in time (visit) in the control group. Intervention i * Visit j is the interaction term, which is the primary parameter of interest. The coefficient β3 quantifies the difference in the time trend between the intervention and control groups (the effect of the intervention over time). The final Xij representing the included covariates (baseline demographics, maternal education, maternal age, infant vaccination for age, maternal dietary intake) (see page 6-7, lines 173-196

**Reviewer point # 5**: How was time parametrized in the model?

**Authors response # 5**: Thank you for helpful points. Time was parametrized in the GEE model using a categorical (factor) variable, denoted as Visits. The Visit variable included three distinct levels, corresponding to the three fixed, scheduled follow-up visits (Visit 1, Visit 2, and Visit 3). In this case, the baseline visit (Visit 1) was set as the reference category for the visit variable. The model estimated 2 separate coefficients for a single visit. Each of these coefficients represents the change in the log-odds of the outcome (the change in the incidence of ARI) at that specific visit compared to the baseline visit, within the control group.

In our analysis, we choose the categorical approach over a continuous approach (modeling time as linear or polynomial) for the following reasons: (1) in this study, time does not impose an assumption of a specific functional form (like linearity or quadratic shape) on the time trend. It allows the effect of time to vary freely and non-linearly between each observation period. (2) Using categorical time allows the intervention visit interaction term to estimate a unique, visit-specific intervention effect (the difference between the treatment and control group's log-odds at each specific follow-up time point). This approach ensures that we accurately capture any non-linear or lagged treatment effects that might occur over the study duration.

**Reviewer point # 6:** Were the subgroups defined a priori? This is described in lines 216-223. Please move these lines to the methods.

Authors’ response # 6: Thank you for your valuable comments. The subgroups were defined a priori (before data analysis). We agree with your suggestion to relocate this information. The description of the subgroup analyses, currently found in lines 216-223 of the manuscript is moved to the methods section (see page, lines 190-196) in the revised manuscript.

**Reviewer point # 7:** There is no ITT population. The mothers were randomized and some of these did not participate. This is some type of completers analysis - as these were the mothers who completed the first study, and agreed to the follow up study. Given that the benefits of randomization do not apply here, the primary analyses should be adjusted for important confounders. Please describe this in the statistical analysis section, and remove references to the ITT analysis.

**Authors response # 7**: Thank you for this critical observation. We accept your comment. We agree that the analysis population for the follow-up study constitutes a completers analysis, not a true Intention-to-Treat (ITT) population, as it only includes mothers who completed the first study and consented to follow-up. Accordingly, we have removed all references to ITT analysis and revised the statistical analysis section to describe the analysis population and the necessary adjustment for confounders (see page 6, lines 173-175) in the revised manuscript).

**Reviewer point # 8**: Were there any twins? How were these handled in the analysis?

**Authors response # 8**: Thank you for your question. There were no twin births in the study. Twin pregnancies were excluded during the initial screening process as they potentially affect birth weight, a key variable in the main trail design. Therefore, no special handling for twins was required during the analysis as stated (see page 4, lines 105-110) in the revised manuscript.

**Reviewer point # 9**: Line 172 - 355 infants are referred to but there were only 343 live births. Please clarify.

**Authors’ response # 9:** Thank you for pointing out this discrepancy. We apologize for the lack of clarity. The reference to 355 infants (Lines 172) pertains to the total number of infants enrolled in the postnatal study who were followed up. However, only 343 infants were ultimately included in the ARI analysis. The difference of 12 infants is due to exclusions (loss to follow up before the first visit, neuronal death and withdrawal of consent). We have revised the manuscript (page 4, lines 114-118) to clearly state the number of infants enrolled in the postnatal follow-up study and those included in the primary ARI analysis. The ambiguous figure '355' has been removed to prevent confusion. The complete randomization and recruitment process for all participants across the parent trial and the subsequent follow-up is fully detailed in Figure 2. More detail statement is also found in the trial profile section (see page 7, lines 201-209)

**Reviewer point # 10**: Line 175 - what is a longitudinal prevalence? Please describe how this was estimated.

**Authors response # 10:** We thank you for raising this important question regarding the precise epidemiological measure used. While the term longitudinal prevalenc**e** is sometimes used broadly to refer to the cumulative occurrence of a condition over a study period, in strict epidemiology, prevalence measures the existing burden (all cases, new and old) at a point in time. Given the design of our Randomized Controlled Trial (RCT), where we followed infants from birth (when they were non-diseased) to six months to track the development of new cases of ARI, the measure we calculated is appropriately termed Cumulative Incidence, not prevalence.

The Cumulative Incidence of ARI was estimated as the proportion of infants who experienced at least one episode of ARI during the entire 6-month follow-up period.

The estimation was calculated using the following formula:

Cumulative Incidence = Number of Infants with at least one ARI episode between 0 and 6 months

Total Number of Infants at Risk at the start of the follow-up (N=355)

This measure reflects the absolute risk an infant had of developing ARI during the first six months of life, based on the assumption that all infants were at risk from the start of the follow-up. To eliminate any confusion, we have replaced the term longitudinal prevalence with cumulative incidence throughout the manuscript, including the statistical analysis and results sections. The paragraph is rewritten in the revised manuscript (page 7, line 190-200)

**Reviewer point # 11**: The statistical analysis section describe incidence rate of ARI, but in the results lines 173-175 prevalence is used. Please clarify.

**Authors’ response # 11:** Thank you for pointing out this issue of clarity. The comment is accepted and correction has been made through the revised document. We confirm that the term 'prevalence' was removed throughout the revised manuscript to ensure consistent terminology

**Reviewer point # 12**: It seems from the data collection that exact time of respiratory infection was collected, but in the analysis, only three time points were used? Please clarify.

**Authors’ response # 12:** That is a very good point, and we appreciate the chance to clarify the data usage. We acknowledge the significant operational challenges inherent in conducting this type of longitudinal study in rural household settings, particularly concerning the accurate capture of morbidity data. To maximize the detection and accurate reporting of ARI incidence in both study arms, we implemented a structured recall-based strategy. Pregnant women were thoroughly trained and instructed to either record or carefully remember the exact day of the incidence onset of any ARI symptoms observed in their infants. This crucial information was then systematically reported to the data collector during their subsequent scheduled household visits. Therefore, while the exact date of the respiratory infection onset was collected during the bi-monthly follow-up visits, we used the three scheduled time points (2, 4, and 6 months) in the primary analysis to assess cumulative incidence and the overall incidence rate for the entire 6-month period. However, the precise onset date was not discarded. It was crucial for person-time calculation and defining recurrence.

**Reviewer point # 13**: Line 199. "GEE model" GEE is not a model. You are using a marginal Poisson model estimated via GEE.

**Authors’ response # 13:** Thank you for your precise methodological distinction. You are correct that Generalized Estimating Equations (GEE) is an estimation method for fitting regression models, not a model itself. We are using GEE to fit a marginal Poisson model. We thank you for this accurate and important methodological clarification.

We accept the comment and recognize that GEE refers to the estimation technique, not the model structure itself. We confirm that our analysis employed a marginal Poisson model to analyze the count data (episodes of ARI), which was estimated using the Generalized Estimating Equations (GEE) approach. We have revised the manuscript to clearly state this in the statistical analysis section (see page 6, lines 179-183) in the revised manuscript.

**Reviewer point # 14**: Lines 199-201 results from a marginal model estimated via GEE and a Poisson model are described. In the stats section, it seemed like these were the same thing. Please clarify.

**Authors’ response # 14:** We appreciate the opportunity to clarify the distinction between the two reported models. While both models use a Poisson distribution appropriate for count data (episodes of ARI), they differ fundamentally in how they handle the correlation resulting from repeated measurements within the same infants over time. The Marginal Poisson Model Estimated via GEE accounts for the within-subject correlation of the ARI episodes (since each infant contributed multiple observations over 6 months). This model provides an Incidence Rate Ratio (IRR) that represents the population-averaged effect. The resulting IRR (0.80) estimates the average effect of the intervention across the entire study cohort, effectively estimating what the difference would be if you randomly selected one infant from each group.

While the Standard Poisson Regression Model does not account for the within-subject correlation of the repeated ARI episodes. It treats each measurement as independent. The resulting IRR (0.82) is an estimate of the intervention effect, but the confidence intervals and p-values may be understated (too narrow or small) because the correlation in the data is ignored, violating the assumption of independent observations.

We reported both results to demonstrate the robustness of the effect size across different modeling assumptions (IRR of 0.80 vs. 0.82). However, as noted in our revised Statistical Analysis section, the marginal Poisson model estimated via GEE is the primary and methodologically superior analysis because it correctly accounts for the repeated nature of our longitudinal data.

**Reviewer point # 15**: Subgroup analyses: In addition to presenting the subgroup specific effects, please include a p value that tests whether the effects differ in the subgroups (e.g. males vs. females).

**Authors’ response # 15:** Thank you for your valuable comment regarding the subgroup analysis presentation. We agree that the Forest Plot graph was not clearly visible and did not comprehensively present the necessary data, particularly the IRR and their respective p-values. To improve clarity and accessibility, we have made a major revision to this section: the results of the subgroup analysis are now presented in a separate, dedicated table (Table 2), which allows for a more precise and comprehensive display of the IRR, confidence intervals, and p -values for each subgroup.

**Reviewer point # 16**: Figure 2 would be more effective as a table with CIs for the IRRs and also showing crude and adjusted IRRs.

**Authors’ response # 16:** Thank you for your suggestion. We interpreted your suggestion as referring to Figure 3 or 4, because Figure 2 is the standard CONSORT flow diagram used to illustrate participant randomization and inclusion. For Figure 3 or 4, Table 2 which appears to have been missed, shows the IRR with confidence intervals for each subgroup.

**Reviewer point # 17**: Line 294 - results described do not match that presented in the Figures female or male infants? Same for abstract.
**Authors’ response # 17:** Thank you for your critical observation. A major revision has been made to the manuscript, and all misplaced values have been corrected throughout the text and tables.

**Reviewer point # 18:** Many results are described in the text of the results section. Please make sure these are also included in tables.

**Authors’ response # 18:** Thank you for raising this point. We can confirm that all results discussed in the text are now fully presented in the tables included in the current version of the document.

**Reviewer point # 19:** There is no Table 1 or 2 - despite being referred to in the text.

**Authors’ response # 19:** We appreciate your concerns. Please accept our apologies. You are correct; Tables 1 and 2 were inadvertently omitted from the original submission. We sincerely apologize for the inconvenience this caused during your review. We have now included the tables in the revised manuscript.

**Reviewer point # 20:** Could you also present results that consider time as a continuous covariate? With a term for intervention and the interaction between time and interaction?

**Authors’ response # 20:** Thank you for your request of time as a continuous covariate. Unfortunately, we are unable to present the results considering time as a continuous covariate with terms for intervention and the time-by-intervention interaction. This decision is based on two primary factors: the first one is due to data collection schedule (discrete intervals). Our study design involves data collection at pre-specified, fixed, and discrete time points (baseline, 3 months, and 6 months), rather than continuously. Treating time as a continuous variable in this scenario, with sparse, distinct measurements, incorrectly assumes a smooth, linear progression between collection points. This assumption risks generating biased or misleading estimates and prevents the model from capturing any non-linear changes or nuances that occur between the fixed measurement times.

The second one is the analytical strategy (categorical approach). Our current analytical strategy, which aligns with the discrete nature of the data, models time as a categorical factor (using separate dummy variables for each follow-up point). Using time as a categorical factor yields a unique estimate of the outcome at each specific follow-up point, accurately capturing non-linear changes over time. Our current approach directly addresses the primary research questions by focusing on comparing the intervention effect (with its confidence interval) at each discrete measurement time. The requested continuous model is typically reserved for instances where the primary goal is to model a rate of change (slope), which is not our focus.

**Reviewer point # 21:** A sensitivity analysis that considers time to first respiratory infection, with time zero being birth and censoring occurring at the end of the study, adjusting for important confounders, using Cox PH regression would be a nice addition.

**Authors response # 21:** We appreciate the your suggestion to perform a sensitivity analysis using a Cox Proportional Hazards (PH) model to assess the time to first respiratory infection (RI), with time zero being birth. While we acknowledge the statistical value of a time-to-event analysis, we must respectfully decline this request for further analysis for two key reasons that relate to the scope and design of our current study: the first one is lack of granular time-to-event data. Our study was fundamentally designed around assessing intervention effects at pre-specified, fixed follow-up points. We did not collect the precise date or time of the first RI occurrence; rather, we recorded whether the event had occurred since the last visit. The available data point is primarily categorical or cumulative within a discrete interval, not the precise time to the event from birth necessary for accurate Cox modeling. Imputing or approximating the event time for the Cox PH model would introduce significant measurement error and potential bias, undermining the validity of the sensitivity analysis itself.

The second is the focus of primary research question. The core objective of our study is centered on comparing the rate of ARI between groups at specific, predetermined clinical milestones (as reflected in our primary categorical analysis), rather than estimating the instantaneous hazard of the event over continuous time. The proposed Cox analysis, while interesting, addresses a different research question (time to event) that requires a distinct data structure and clinical focus than our current manuscript. Implementing it would necessitate making assumptions that violate the integrity of the data collected under the current study design.

Given these limitations, we believe the added analytical complexity and the risk of bias due to insufficient data granularity outweigh the benefit of this supplementary analysis. Our current analytical approach, which models time categorically, remains the most appropriate and robust method for our existing dataset.

**Reviewer #2**

This randomized controlled trial examined whether providing locally made, chimney-fitted improved stoves (Mirt stoves) to pregnant women in rural Ethiopia could reduce the incidence of acute respiratory infections (ARIs) in their infants during the first six months of life. A total of 343 infants were monitored, with respiratory symptoms assessed every two months. The results showed that 12% of infants experienced at least one ARI episode, with a slightly lower incidence in the intervention group (10.7%) compared to the control group (13%). However, this difference was not statistically significant. The intervention group also showed a 19-20% lower risk of ARI, but again, this reduction did not reach statistical significance.

Subgroup analyses suggested possible benefits for female infants and those from larger families, but these trends were also not statistically significant. Importantly, indoor air pollution levels remained much higher than World Health Organization guidelines in both groups, which may have limited the health impact of the stove intervention. These findings align with other research in similar settings, where improved stoves often reduce household air pollution but do not consistently lower ARI rates in young children. The study concludes that while improved stoves may have some protective effect, larger and longer-term studies are needed to confirm these trends and better understand their impact on child respiratory health.

I appreciate the important contribution your study makes to understanding the impact of improved stoves on child respiratory health. I have several suggestions that I believe could strengthen your manuscript:
**Reviewer point # 1:** Clarify and Streamline the Abstract: I recommend stating the primary outcome and key findings more explicitly in the abstract, for example, noting that no statistically significant reduction in ARI incidence was observed, but a non-significant trend toward benefit was noted. I also suggest shortening and focusing the abstract to better highlight the study design, main results, and implications.

**Authors response # 1:** We appreciate your excellent suggestion to enhance the clarity and focus of the abstract. We agree that stating the primary outcome and key findings more explicitly will significantly improve the abstract's impact and readability. We have revised the abstract based on your suggestions to be more concise and explicit (see page 2, lines 40-59)

**Reviewer point # 2**: Improve Reporting of Statistical Results: I would find it helpful if you reported absolute numbers alongside percentages for key outcomes (e.g., "43 infants [12%] experienced at least one ARI episode"). Additionally, clarifying the meaning of IRR, CI, and p-values in lay terms would make the results more accessible. Providing effect sizes and confidence intervals for subgroup analyses in a concise table or summary would also be valuable.

**Authors’ response # 2:** We greatly appreciate your detailed suggestions for improving the reporting of our statistical results. We agree that adopting these changes will significantly enhance the clarity, accessibility, and transparency of our findings. We ensured that absolute numbers are consistently reported alongside percentages for all key outcomes in both the abstract and the main text in the revised manuscript. To concisely and transparently present the subgroup findings, we will add a concise table or figure to the Results section. This table will systematically display the effect sizes (IRR) and 95% Confidence Intervals (CI) for the primary outcome across all relevant subgroups. This approach will prevent clutter in the main text while providing necessary detail.

**Reviewer point # 3**: Enhance Methodological Detail: I suggest briefly explaining the rationale for using Generalized Estimating Equations (GEE), as some readers may be unfamiliar with this method. Please clarify the definition of ARI, specifying which symptoms were included and how they were assessed. It would also strengthen the manuscript to describe how missing data, dropouts, and losses to follow-up were handled in your analysis.

**Authors response # 3:** We appreciate your valuable comments and suggestions. The rationale for utilizing Generalized Estimating Equations (GEE) has been included in the data analysis section (see page 6, lines 179-183). We selected GEE to address the longitudinal design of the study. Specifically, we employed a marginal Poisson regression model, estimated via GEE, to formally account for the correlation arising from the repeated measurements taken within the same infant over the 6-month follow-up period. GEE provides robust standard error estimates and allows us to estimate the average change in the risk of Acute Respiratory Infection (ARI) over time while appropriately handling the non-independence of repeated observations from the same infant. The definition of Acute Respiratory Infection (ARI) used in this study, detailed in the outcome assessment section ( see page 5, lines 147-149), is a mother-reported syndrome based on specific symptoms and duration. An ARI episode was defined as the mother reporting symptoms that met three criteria: 1) symptoms were present for at least two consecutive days; 2) at least two symptoms were reported on at least one day; and 3) at least one symptom was present on each day. The symptoms included in the definition were cough, difficulty breathing, and runny/stuffy nose.

We excluded participants who were lost to follow-up before the first visit, experienced neonatal death, or refused to participate. After accounting for all subsequent dropouts, a total of 343 infants completed the full study follow-up. For the primary analysis, we employed an available case approach by only considering the complete data available for each visit, as we did not impute missing data. The choice of Generalized Estimating Equations (GEE) inherently addresses data attrition in longitudinal studies. GEE provides robust estimates even when data are missing at random, as it utilizes all available measurements from all participants up to the point of dropout. This robustness makes full imputation unnecessary for our primary analysis, provided the data loss mechanism is missing at random or missing completely at random (see page 5, lines 148-150).

**Reviewer point # 4**: Address Potential Confounders and Bias: I encourage you to explicitly discuss how differences in maternal age and vaccination status were controlled for in adjusted analyses. Please also mention any steps taken to minimize reporting bias in symptom reporting by mothers.
**Authors’ response # 4:** Thank you for your helpful suggestions**.** We agree that controlling for potential confounders is crucial for enhancing the validity of our findings. Maternal age was explicitly included in the multivariable analysis as a covariate in the Generalized Estimating Equations (GEE) model. This adjusted analysis allowed us to estimate the independent association between our primary exposure and the outcome (ARI risk) while accounting for the effect of maternal age. Infant vaccination status (specifically adherence to the routine immunization schedule for age, which is a key determinant of infant health) was also included as a categorical confounder in the adjusted GEE model. This ensures that the risk of ARI is estimated while accounting for the protective effects or differences associated with the timing of vaccine uptake across the follow-up period.

We acknowledge the potential for reporting bias since the outcome (ARI episodes) relies on mother-reported data. We took the following steps to minimize this bias. (1) The study employed a strict, multi-criterion definition of ARI (at least two consecutive days, with specific symptom counts) to objectify the subjective reporting. (2) Field data collectors were thoroughly trained to ensure the symptoms were assessed and recorded uniformly across all mother-infant pairs. (3) Data collection occurred during routine, predetermined follow-up visits, rather than solely when the infant was acutely ill. This approach helps to mitigate recall bias and ensures that symptom reporting is captured consistently over time, regardless of the severity of a current episode. Data collectors were trained to maintain a neutral approach, emphasizing the importance of accurate reporting of all symptoms (or lack thereof) rather than implicitly encouraging the reporting of illness.

**Reviewer point # 5**: Strengthen the Discussion: I would appreciate a more direct comparison of your findings with similar studies (such as those from KidsAir, Rwanda, and Mexico), including possible reasons for similarities or differences. Discussing the implications of persistently high PM2.5 levels despite the intervention, as well as the public health relevance and practical challenges of stove interventions in rural settings, would add valuable context.

**Authors’ response # 5:** We appreciate your suggestion to strengthen the discussion section. We have thoroughly revised the discussion to incorporate a more direct and detailed comparison of our findings with similar studies, along with a focused discussion on the implications of the intervention's effectiveness (see page 10, lines 288 -306). The revised discussion now includes one dedicated paragraph on the public health relevance of our findings. Furthermore, we address the practical challenges of stove interventions in rural settings, highlighting key factors that (see page 13, lines 362- 374)

**Reviewer point # 6:** Improve Figures and Tables: Please ensure that all figures and tables are clearly labeled and referenced in the text. I also suggest adding a summary table of key outcomes (incidence, IRR, CI, p-value) for quick reference.

**Authors’ response # 6:** Thank you for raising the concern regarding figure and table labeling. We have confirmed that all figures are now clearly and appropriately labeled, and the requested summary table is included in the revised manuscript (see Table 2).

**Reviewer point # 7:** Refine Language and Structure: I recommend editing the manuscript for clarity and conciseness, especially in the introduction and discussion sections. Removing repetition and using consistent terminology (e.g., always referring to the improved stove as "Mirt stove" or "improved stove") would enhance readability.

**Authors’ response # 7:** Thank you for your valuable feedback regarding the manuscript's clarity, conciseness, and terminology. We agree that refining the language and structure will significantly enhance the readability of the paper. We have addressed these points throughout the revised manuscript. We have meticulously edited the introduction and discussion sections to eliminate redundancy and strengthen sentence construction, resulting in a more direct and concise narrative. We have established and maintained consistent terminology throughout the entire manuscript. Specifically, we now exclusively refer to the intervention technology as the "improved stove" to ensure clarity and avoid ambiguity.

**Reviewer point # 8:** Address Limitations More Explicitly: I believe it would be helpful to more clearly acknowledge the study's limited power to detect small differences due to sample size and follow-up duration. Discussing potential generalizability issues, such as unique features of the Ethiopian setting or cultural practices, would also be beneficial.

**Authors’ response # 8:** Thank you for the constructive feedback regarding the limitations section. We agree that a more explicit acknowledgement of statistical power, sample size constraints, and generalizability issues will strengthen the study's interpretation. We have revised the limitations section of the discussion to more clearly address these points (see page 13, lines 375-384)

**Reviewer point # 9:** By addressing these points, I believe your manuscript will be clearer, more rigorous, and more compelling for both scientific and policy audiences. Thank you for your valuable work in this important area.

**Authors response # 9:** We appreciate your final positive assessment and your guidance on strengthening the manuscript. We hoped that we have addressed all the suggested points regarding clarity, rigor, and compelling presentation. We also believe these comprehensive revisions have successfully made the manuscript clearer, more rigorous, and more compelling for both the scientific community and policy audiences. Thank you again for your time and thoughtful review of our work.

**Reviewer # 3**

**Reviewer Point:** Your article PONE-D-25-24436 is well-written, and the scientific concept is strong. However, it requires further investigation with a larger sample size and a longer follow-up period. Additionally, the levels of PM2.5 in both the intervention (104 μg/m³) and control (449 μg/m³) household kitchens after the intervention significantly exceed the World Health Organization's recommended safe limits. This is an important point to consider when comparing with other studies.
The similarity report, which is attached, shows a similarity index of 33%, and I believe there is room for improvement in this area. Furthermore, the acronym HAP is first mentioned in line 74 without prior explanation. Additionally, in line 288 ARI, it is mistakenly referred to as ALRI.

**Authors’ response # 8:** We appreciate your positive assessment of our manuscript’s scientific concept and writing quality. We acknowledge that a larger sample size and longer follow-up period would strengthen the study's power. We have explicitly addressed this as a limitation in the revised manuscript, acknowledging that the study may be underpowered to detect smaller effects. We have enhanced the discussion section to explicitly address the implications of the persistently high PM2.5 levels (Intervention: 104ug /m3; Control: 449 ug/m3), noting that both significantly exceed WHO safe limits. We now discuss how this context likely contributes to the non-significant health outcomes and must be considered when comparing our results to other interventions. The acronym HAP (Household Air Pollution) is now defined at its first mention in the introduction. We have also defined acute lower respiratory infection (ALRI) at its first mention and is used consistently. We believe these revisions satisfy all concerns, strengthening the rigor and readability of the manuscript.

**Reviewer # 4**

**Reviewer point #1:** Line 71: HAP is not defined before
**Authors response # 1:** Thank you for your comment. The suggested correction has been accepted and implemented (see page 3, Lines 73–74).

**Reviewer point #2:** Line 83-85: I understood the gap. But stating the goal of this study is missing. Few sentences could be added to explain the goal and objectives of this research.

**Authors response # 2:** Thank you for pointing out the missing clarity regarding the study's goal following the identified gap. We agree that the manuscript will be strengthened by explicitly stating the research objectives at the end of the introduction. We have revised this section of the introduction by adding sentences to explicitly state the goal and objectives of the research (see page 3, lines 88-90)

**Reviewer point #3:** Line 92: citations [30, 31]. I would prefer a summary or gist from those articles. Simply mentioning to check the articles may fail to describe the intention.

**Authors’ response # 3:** Thank you for the comment. We included more details about the study area in the revised manuscript (see page 4, lines 91-101)

**Reviewer point #4:** Line 97: Provide a little bit summary from that study.

**Authors response # 4:** The comment was accepted and correction has been done (page 4, lines 105-110)

**Reviewer point #5:** Line 110: Picture resolution is poor

**Authors response # 5:** Thank you for your concern regarding the figure quality. We have improved the resolution of all figures in the revised manuscript.

**Reviewer point #6:** Line 110: Initially Mirt was written in italic form in line 103 but it is no longer italic in line 110

**Authors response # 6:** Thank you for your comment. We have accepted the suggestion and removed the italic formatting in line 110 of the revised manuscript.

**Reviewer point #7:** Line 139: Have any other studies used this method to explore similar or relevant topics? If yes, there should be some examples.

**Authors’ response # 7:**

**Reviewer point #8:** Line 139: Why did you use Poisson regression with generalized estimating equations? Explanation is missing. Why not other methods? What are most commonly used methods? Why this one differs from all other methods?

**Authors response # 8:** Thank you for requesting clarification on our choice of statistical modeling. We agree that a more detailed justification for using Poisson regression with Generalized Estimating Equations (GEEs) is warranted. We employed Poisson regression with Generalized Estimating Equations (GEEs) for the following reasons: (1) Nature of the Outcome Variable: The primary outcome, Acute Respiratory Infection (ARI) incidence, is a count outcome (number of ARI episodes per child per period). Poisson regression is the standard choice for modeling count data, providing the appropriate Incidence Rate Ratio (IRR) as the measure of effect. (2) Longitudinal/Correlated Data Structure: Our study utilizes a longitudinal design with multiple ARI measurements collected from the same infant across repeated post-natal visits. This design inherently leads to within-subject correlation (i.e., the ARI rate measured at one visit is not independent of the rate at the next visit for the same child). (3) GEE Advantage: The GEE approach is specifically designed to account for this non-independence (correlation) in clustered or longitudinal data. Unlike standard Poisson regression or Mixed-Effects Models (which model the dependence structure of the subject), GEE focuses on providing robust and consistent estimates of the population-averaged effect (the IRR). This method is well-suited for randomized controlled trials where the primary interest is the overall effect of the intervention across the entire study population.

**Reviewer point # 9:** Line 143: Rephrase

**Authors’ response # 9:** Thank you for your critical observation. We have rephrased the sentence and corrected the double written word 'subgroup' in the revised manuscript.

**Reviewer point #10:** Line 144: Why the mentioned variables are important to study?

**Authors’ response # 10:** Thank you for requesting clarification on the selection of subgroup variables. The mentioned variables were included to investigate potential effect modification and identify whether the intervention's effectiveness varied among populations known to have different baseline risks or exposure levels to Acute Respiratory Infections (ARI). Some of the justification for including each variable in the subgroup analysis are;

**Newborn Sex:** Biological differences (airway size, immune response) and behavioral factors are sometimes associated with varying susceptibility to ARI in infants, making it important to test for differential intervention effect.

**Gestational Age:** Infants born preterm (<37 weeks) often have underdeveloped lungs and immune systems, placing them at a significantly higher risk for severe ARI. The intervention's effectiveness might therefore be magnified or diminished in this high-risk group.

**Kitchen Roof Type:** This variable (thatched vs. CIS/corrugated iron sheet) serves as a proxy for kitchen ventilation and air exchange rates. Poor ventilation (often associated with certain roof types) increases pollutant concentration, potentially modifying the intervention stove's benefit.

**Family Size:** A larger family size (more occupants) increases the risk of infectious disease transmission and may be related to housing density and overcrowding, which are known risk factors for ARI incidence, independent of household air pollution (HAP). Large family size also demands large volume of meal which needs prolonged cooking that exposures to high level pollutant for long time.

**Exclusive Breastfeeding:** Breastfeeding is a crucial protective factor, significantly boosting the infant's immune system and reducing the risk and severity of respiratory infections. It's essential to see if the stove intervention adds benefit over and above this strong biological protection.

**Vaccination Status:** Vaccination status (specifically, for age) is another measure of baseline immunity and parental adherence to preventative health measures. This variable helps assess if the intervention's impact differs between infants who already have high protection against common infectious agents and those who do not.

**Reviewer point #11:** Line 156-157: Check spacing

**Authors’ response # 11:** Thank you for the comment. The space is checked and corrected

**Reviewer point #12:** Line 257: citation bracket is in red color

**Authors’ response # 12:** Thank you again for your critical observation. Correction has been made.

**Reviewer point #13:** Line 262-263: spacing between paragraphs

**Authors’ response # 13:** Thank you, the paragraph spacing is corrected in the revised manuscript.

**Reviewer point #14:** Line 293-294: spacing

**Authors’ response # 14:** We appreciate your detail observation. Correction has been made in the revised manuscript.

**Reviewer # 5**

**Sample Size**
 **Reviewer point #1:** No power calculation (sample size) for the study- the authors just mentioned that they followed 343 infants. So the study could be underpowered or overpowered.

**Authors’ response # 1:** Thank you for raising the crucial point regarding the absence of an explicit power calculation in the manuscript. We acknowledge this oversight. The initial sample size was calculated for the primary cohort (pregnant women) recruited for the main trial based on standard power calculations designed to detect a minimum clinically relevant difference in the primary outcome. Subsequently, all live-birth infants born to this cohort were followed for the secondary outcome (ARI development), ensuring all available subjects were included in this analysis. The detailed justification for the sample size, including the parameters used and the minimum detectable difference, has been added to the revised Methods section of the manuscript. (For your convenience, these details were also provided in our direct response to Reviewer 1, Question #1).

**Statistical Analysis**
**Reviewer point #2:** It is not clear what type of correlation structure was used in GEE.

**Authors’ response # 2:** Thank you for requesting additional detail regarding the Generalized Estimating Equations (GEE) approach. We agree that these specifics are essential for reproducibility and transparency, and we have fully elaborated on these details in the statistical analysis section of the revised manuscript.

The GEE model was fitted using an Exchangeable Working Correlation Structure. This structure was chosen because our analysis involves repeated measurements of the ARI outcome taken at fixed, scheduled intervals (visits) across all participants. The exchangeable structure simplifies the analysis by assuming that the correlation between any two measurements taken on the same infant is constant, regardless of the time interval separating them. While we acknowledge that an ARI structure might also be plausible for time-based data, the Exchangeable structure offered a more parsimonious fit and did not significantly alter the primary inference compared to more complex structures.

**Reviewer point #3:** Table 1 (baseline data), Table 2 (results table) mentioned in the text, but I didn’t see any such table. It is not possible to complete the review.

**Authors’ response # 3:** Thank you for bringing to our attention that Table 1 (Baseline Data) and Table 2 (Results Table) were referenced in the text but were missing from the manuscript file you received. We apologize for this significant oversight. We confirm that both Table 1 and Table 2 are essential for completing the scientific review. We have now ensured that both tables are correctly attached and included in the revised manuscript file according to the journal's formatting guidelines. We believe the presence of these tables will allow you to complete your review of the data and findings.
